# Intestinal Colonization by *Campylobacter jejuni*, *Clostridium difficile*, and *Clostridium perfringens* among Commensal *Rattus norvegicus* in the Urban Areas of Tehran, Iran

**DOI:** 10.1155/2024/2929315

**Published:** 2024-03-27

**Authors:** Taher Azimi, Sina Nasrollahian, Sahar Sabour, Mehrdad Mosadegh, Nahal Hadi, Leila Azimi, Fatemeh Fallah, Mohammad Reza Pourmand

**Affiliations:** ^1^Department of Bacteriology & Virology, School of Medicine, Shiraz University of Medical Sciences, Shiraz, Iran; ^2^Department of Microbiology, Faculty of Medicine, Ahvaz Jundishapur University of Medical Sciences, Ahvaz, Iran; ^3^Department of Pathobiology, School of Public Health, Tehran University of Medical Sciences, Tehran, Iran; ^4^Pediatric Infections Research Center, Research Institute for Children's Health, Shahid Beheshti University of Medical Sciences, Tehran, Iran

## Abstract

**Background:**

*Rattus norvegicus* (*R. norvegicus*) population plays a significant role in the spread of numerous diseases in urban environments. The present study is aimed at investigating the presence of *Campylobacter jejuni* (*C. jejuni*), *C. coli*, *Clostridium difficile* (*C. difficile*), *C. difficile* toxigenic, and *C. perfringens* in *R. norvegicus* captured from urban areas of Tehran, Iran.

**Methods:**

From October 2021 to October 2022, 100 urban rats were trapped in 5 different districts of Tehran, Iran. The genomic DNA was extracted from fecal samples, and the presence of *C. jejuni*, *C. coli*, *C. perfringens*, and *C. difficile* species was evaluated using PCR assay. Moreover, PCR was used to assess the toxicity of *C. difficile* isolates.

**Results:**

Overall, 30% (*n* = 30/100) of fecal samples were positive for zoonotic pathogens. Based on the PCR on hippuricase (*hipO*), glycine (*gly*), *CIDIF*, and phospholipase C (*plc*) genes, *C. perfringens* and *C. difficile* were isolated from 18.2% (*n* = 14/77) and 5.2% (*n* = 4/77) of male rats. The highest frequency of *C. perfringens* and *C*. *jejuni* was 25% (*n* = 5/20) related to the south of Tehran. Toxigenic *C. difficile* was not detected in all regions.

**Conclusion:**

According to the findings, rats are the main reservoirs for diseases. Therefore, rodent control coupled with the implementation of surveillance systems should be prioritized for urban health.

## 1. Introduction

All recently discovered severe infectious diseases in people are brought on by zoonotic pathogens. Even though a variety of factors, including interactions between animal reservoirs and the population at large, affect zoonotic transmission [1, 2], events that act to lessen the ecological or physical distance between human and animal populations or to increase the density and abundance of these groups where they coexist raise the risk of zoonotic transmission [3].

The Norway rat (*Rattus norvegicus*) is a known zoonotic pathogen reservoir [4]. They can be found everywhere, both in urban and rural areas. These rodents share a home with people, eat human waste, and interact with many components of the food supply while residing in buildings [5].

It is well known that *R. norvegicus* is a significant source of disease for humans and can transmit a variety of different pathogens through direct contact and contamination of food items, or the environment. They are also consistent with high levels of fecundity, growth rates, and population densities [6, 7].

The most common cause of campylobacteriosis in humans is *Campylobacter jejuni* (*C. jejuni*). The most frequently reported zoonotic disease in the urban region is caused by *Campylobacter* spp., particularly *C. jejuni* and *C. coli*, which are common causes of gastroenteritis in people, worldwide [8]. It has been demonstrated that several rodent species, including the brown rat (*R. norvegicus*), yellow-necked mouse (*Apodemus flavicollis*), house mouse (*Mus musculus*), and bank vole (*Myodes glareolus*), carry *Campylobacter* species in their intestinal tracts [9, 10]. Therefore, wild rats are the reservoir or source of *Campylobacter* spp. for both livestock and people [11].

Gram-positive sporogenic anaerobic bacteria *Clostridium perfringens* (*C. perfringens*) and *Clostridium difficile* (*C. difficile*) are known to cause intestinal illness in both humans and animals. *C. difficile* produces incredibly resilient spores that can survive for a very long time in the environment, aiding in transmission. Numerous animal species have the potential to develop clinical diseases as a result of infection or to become colonized with *C. difficile* [12]. The strains that can infect both humans and animals frequently overlap, making it difficult to identify between human and animal isolates. This has increased the chance of zoonotic *C. difficile* transmission between them through direct contact with the food chain (i.e., tainted meat products) or the environment [13]. The present study is aimed at determining the presence of *C. jejuni*, *C. coli*, *C. difficile*, *C. difficile toxigenic*, and *C. perfringens* in *R. norvegicus* captured from urban areas of Tehran, Iran.

## 2. Materials and Methods

### 2.1. Study Design, Rat Trapping, and Sample Collection

In the present study, 100 *R. norvegicus* were collected from five districts (north, south, west, east, and center) of Tehran, Iran. The sampling plan was designed to capture 20 rats from each district between October 2021 and October 2022. During the peak of their activity in all four seasons, rats were caught using Sherman and Tomahawk professional live traps (Tomahawk Live Trap, Hazelhurst, WI), which had been baited with enticing baits including sunflower seeds and peanut/sesame butter. In general, trapping was done after dusk in each chosen region and was processed at midnight or the next morning, taking into account Tehran Municipality's physical and chemical intervention that was intended to suppress rats. Over several days, fecal samples were collected and stored at -80°C. The following bacteriological test was conducted at Tehran University of Medical Sciences School of Public Health's Department of Pathobiology, Division of Medical Microbiology. The collected rats were then sent to a secure animal housing laboratory and euthanized by excessive isoflurane anesthesia, followed by bilateral thoracotomies.

### 2.2. Sample Transfer and DNA Extraction

All fecal samples were placed in Cary-Blair transport media (Micro Media, Hungary), and they were then transported right away to the microbiological lab. The whole genomic DNA was extracted from all faeces using the AllPrep DNA Mini Kit (Qiagen, Inc.) directly, according to the manufacturer's instructions. Each DNA sample was then eluted in 200 ml of buffer and stored at -80°C until further use.

### 2.3. Molecular Detection of Bacterial Marker Genes

The specific primers for hippuricase (*hipO*), glycine (*gly*), *CIDIF*, and phospholipase C (*plc*) genes were used for the identification of *C. jejuni*, *C. coli*, *C. difficile*, and *C. perfringens*, respectively. The polymerase chain reaction (PCR) was optimized using the four-pair primer sets listed in Table 1. The PCR amplification was settled in 25 *μ*l reaction volumes on a thermal cycler (Eppendorf Mastercycler Gradient, Eppendorf, Hamburg, Germany). The reaction mixture contained 10 *μ*l master mix (Amplicon, Denmark), 0.5 *μ*M of each primer, 1 *μ*l DNA template, and 10 *μ*l distilled water. The optimal PCR cycles and thermal conditions are mentioned in Table 1. PCR products were screened on a 1–1.5% agarose gel, visualized by DNA-safe stain (SinaClon Co., Iran), and photographed under UV light.

### 2.4. Toxin Gene Detection

Using PCR assay, the presence and frequency of the toxin A (*tcdA*), toxin B (*tcdB*), and binary toxin (*cdtA/B*) genes were evaluated in all isolates recognized as *C. difficile*. The sequence of primers used for PCR assay is shown in Table 2. The PCR reactions were done in a total volume of 25 *μ*l. The reaction mixture was described previously. The PCR reactions consisted of an initial denaturation step at 95°C for 5 min; followed by 30 cycles of 60 sec at 95°C; annealing for 45 sec at 51°C (for *tcdA*), 50°C (for *tcdB*), and 53°C (for *cdtA/B*); and extension for 50 sec at 72°C. A final extension step was performed at 72°C for 5 min. The PCR products were separated by electrophoresis on 1.5% agarose gels. *C. perfringens* strain ATCC 13124 and *C. difficile* strain, which were sequenced and confirmed in previous studies in the Department of Microbiology, Faculty of Health, Tehran University of Medical Sciences, were used as positive controls in the reaction.

### 2.5. Statistical Analyses

The statistical program SPSS v.23.0 (SPSS Inc., Chicago, IL, USA) was used to evaluate the data using descriptive statistic tests after the data were formatted in an SPSS file.

## 3. Results

From October 2021 to October 2022, a total of 100 *R. norvegicus* were captured in five districts of Tehran, Iran. 30% (*n* = 30/100) of the samples collected from rats were positive for different pathogens. The majority of rats were male with 77% and 23% being female. According to the positive results of *hipO*, *Gly*, *CIDIF*, and *Plc*, *C. perfringens* (18.2%; *n* = 14/77 versus 4.3%; *n* = 1/23) and *C. jejuni* (14.3%; *n* = 11/77 versus 0%; *n* = 0/23) had the highest frequency among male and female rats, respectively.

The highest frequency of pathogens was found in the south of Tehran (25%; *n* = 5/20), but *C. coli* and *C. difficile* were not detected in all regions (Table 3). Toxigenic *C. difficile* was not detected in all regions.

## 4. Discussion

*R. norvegicus* populations are significant players in the ecology of zoonotic diseases and carry zoonotic pathogens in urban settings [14]. They are particularly well-adapted to residing close to places where people live, and they may result in significant economic losses by damaging materials, consuming and defecating on food sources, and transmitting illness [15]. Implementing control measures will be much easier if the epidemiology and ecology of the pathogenic agent are better understood. Several nations have established health surveillance programs to manage human campylobacteriosis. Campylobacteriosis was not widely reported in the United States until 2015. Nevertheless, up until that point, the states decided to notify the National Notifiable Diseases Surveillance System of any new cases [16]. Due to the difficulties in detecting *Campylobacter* in developing nations, accurate information on the prevalence of infection with these bacteria is typically not given. The *Campylobacter* was isolated and identified using techniques including culture on certain media. These procedures, however, require more manual labor and are less accurate than serological and molecular approaches. Another possibility is that under unfavorable circumstances, *Campylobacter* is alive but unculturable, leading to erroneous negative results [8].

The present study is aimed at investigating the presence of *C. jejuni*, *C. coli*, *C. difficile*, *C. difficile* toxigenic, and *C. perfringens* in *R. norvegicus* captured from urban areas of Tehran, Iran. Our results revealed that *C. perfringens* was the main pathogen that was frequently isolated from the *Rattus* population of Tehran. In addition, the frequency of *C. perfringens* was high in the south (25%), west (20%), and center (20%) of Tehran. This finding illustrates that *C. perfringens* is the main gastrointestinal pathogen in the *Rattus* population, and these rodents are the reservoir of this pathogen. In a study conducted in 2022 by Milton et al., *C. perfringens* was isolated from 27 out of 122 fecal samples collected from rodents [17]. Their study's findings are comparable to ours in that *C. perfringens* (18.2% of the male rats) was the most often investigated pathogen. *C. perfringens* was recovered from 26 fecal samples from *R. norvegicus* (76.5%) in 2014, according to Silva et al. There was no significant correlation between *C. perfringens* isolation and length of confinement [18]. According to a study by Firth et al., 133 Norway rats were collected in 2014 from five different areas of New York City. The majority of infections among male rodents have similar frequencies to our findings [19]. According to recent studies, probably the reason why the frequency of pathogens is higher among male rats is because males compete with each other to mate with females, and during this competition, they get injured, through this, the pathogen enters the male rat's body [20].

Based on several reasons such as (1) the high prevalence of rodents in urban environments, (2) their enormous influence on human health, and (3) the high prevalence of community-associated diseases and zoonotic infections, the presence of rodent populations is a main concern in urban settings [21].

In a study performed by Shahrokhabadi et al., in Iran, *C. jejuni* and *C. coli* were found in 82.35% and 17.65% of the sheep meat samples tested positive for *Campylobacter* spp. [22]. Rahimi et al. earlier stated that *Campylobacter* was present in only 6% of sheep meat [23]. While *Campylobacter* spp. were found in 30% of sheep faeces in Algeria, Guessoum et al. reported finding *Campylobacter* spp. in 3.5% of sheep stool samples in Brazil [24, 25].

The study's findings showed that although it does not appear very plausible that humans could contract a disease from cattle and vice versa, it is crucial to follow good hygiene practices.

Our findings indicated that the frequency of *C. difficile* was 4%, and most of them were related to the center of Tehran. In studies conducted by Byers et al. in 2021, de Oliveira in 2018, and Silva et al. in 2014, *C. difficile* was detected in rats trapped in different regions, but in contrast with our investigation, all of them had toxin genes [7, 11, 12, 18]. However, considering the nontoxicity of the isolated bacterium, it seems that it is an environmental bacterium and does not have much value in terms of causing a significant disease in humans. Also, according to Himsworth et al., the odds of being *C. difficile* positive decreased with increasing weight. Furthermore, it seems that it is more common in young mice with more physical activity and less weight than other older mice [21]. In contrast with this study, we did not evaluate the rats based on weight. Due to the high expense of treatment and healthcare, campylobacteriosis is a more unpleasant intestinal disease that is significant for people and society's health system. It was proven that campylobacteriosis is still monitored in wealthy nations. According to our results, *C. jejuni* was positive in five regions of Tehran. The high frequency is related to the south zone (25%). In a study conducted by Nkogwe et al. in 2011, the frequency of *C. jejuni* among *R. norvegicus* population was 3.4% which was less than our findings [11]. The frequency of this pathogen in Milton et al. was 4%, which demonstrated that depending on the geographical location, the amount of abundance varies, and it is one of the most important pathogens that is mainly carried by rats [17]. In a research performed by Olkkola et al., 20% of the pooled *R. norvegicus* samples from Finnish farms included *C. jejuni* [9]. Additionally, prior research has demonstrated that 9% of *R. norvegicus* samples from pig farms, poultry farms, and nonfarm areas in Sweden contained *Campylobacter* spp., *C. jejuni*, and *C. coli*, respectively [26]. According to research, 40% of the *R. norvegicus* collected from pig farms in France had *C. jejuni* in them [27]. Another investigation found that 12.5% of *R. norvegicus* captured on a Dutch pig farm contained *C. coli* [28]. The limitations of the current study are not examining *Campylobacter* spp. genotypes and evaluating all important pathogens due to a lack of time and budget, and another reason is the small number of rodents in each geographical region in Tehran.

## 5. Conclusion

The findings demonstrated that urban rats were the primary reservoirs that contributed significantly to the spread of zoonotic infections carried by vectors to humans. The risk of acquiring these diseases through the *Rattus* population needs to be brought to the general public's attention. The creation of effective surveillance plans and intervention strategies depends on knowledge of the zoonotic parasites carried by the *R. norvegicus* population in Tehran Province. Further research on other infections in urban rats and other domestic and wild animals in Tehran, Iran, is urgently needed, as these data also show.

## Figures and Tables

**Table 1:** Sequences of primers, cycles, and optimum thermal conditions of PCR.

Marker	Primer sequence (5′->3′)	Amplicon size	PCR condition	Reference
*gly*	F: GCGTCTGTTGACTGGCAGGTGGTGG	121 bp	95°C (7 min) {95°C (5 min), 60°C (45 seconds), 72°C (1 min)} × 35	This study
	R: GTTGCCCGCTTCGAAACCAATGCCT			
*hipO*	F: CTTTTTGCAGACATTGCACACAT	323 bp	95°C (7 min) {95°C (5 min), 55°C (1 min), 72°C (1 min)} × 35	
	R: CGCCTCTTGGACCACGTAAG			
*cidif*	F: CTTGAATATCAAAGGTGAGCCA	1088 bp	95°C (5 min) {95°C (5 min), 55°C (45 seconds), 72°C (1 min)} × 35	
	R: CTACAATCCGAACTGAGAGTA			
*plc*	F: CGTTGATAGCGCAGGACATG	244 bp	95°C (5 min) {95°C (5 min), 58°C (45 seconds), 72°C (1 min)} × 35	
	R: TACCTTTGCTGCATAATCCCAAT			

**Table 2:** Sequences of primers of *C. difficile*'s toxin genes.

Genes	Primer sequence (5′->3′)		References
*tcdA*	F	5′-CAACACCTTAACCCAGCCATA-3′	This study
	R	5′-AGAGTTTTCTGCGGTAGCTGA-3′	
*tcdB*	F	5′-ATCTGGAGAATGGAAGGTGGT-3′	
	R	5′-TGATGGTGCTGAAAAGAAGTG-3′	
*cdtA/B*	F	5′-TATATTAAAGCAGAAGCATCTGT-3′	
	R	5′-CTGGACCATTTGATATTAAATAATT-3′	

**Table 3:** The frequency of zoonotic pathogens identified by PCR in five districts of Tehran.

Frequency	*C. coli* no. (%)	*C. jejuni* no. (%)	*C. difficile* no. (%)	*C. difficile toxigenic* no. (%)	*C. perfringens* no. (%)
Male (77)	0	11 (14.3)	4 (5.2)	0	14 (18.2)
Female (23)	0	0	0	0	1 (4.3)
North (20)	0	1 (5)	1 (5)	0	1 (5)
South (20)	0	5 (25)	0	0	5 (25)
East (20)	0	1 (5)	0	0	1 (5)
West (20)	0	1 (5)	0	0	4 (20)
Center (20)	0	3 (15)	3 (15)	0	4 (20)
Total	0	11	4	0	15

## Data Availability

All data generated or analyzed during this study are included in this published article.
